# First person – Andreas Enström

**DOI:** 10.1242/bio.059642

**Published:** 2022-10-21

## Abstract

First Person is a series of interviews with the first authors of a selection of papers published in Biology Open, helping researchers promote themselves alongside their papers. Andreas Enström is first author on ‘ RGS5: a novel role as a hypoxia-responsive protein that suppresses chemokinetic and chemotactic migration in brain pericytes’, published in BiO. Andreas is a PhD student in the lab of Gesine Paul at Lund University, Sweden, investigating cellular adaptations to fluctuations in oxygen and their implications.


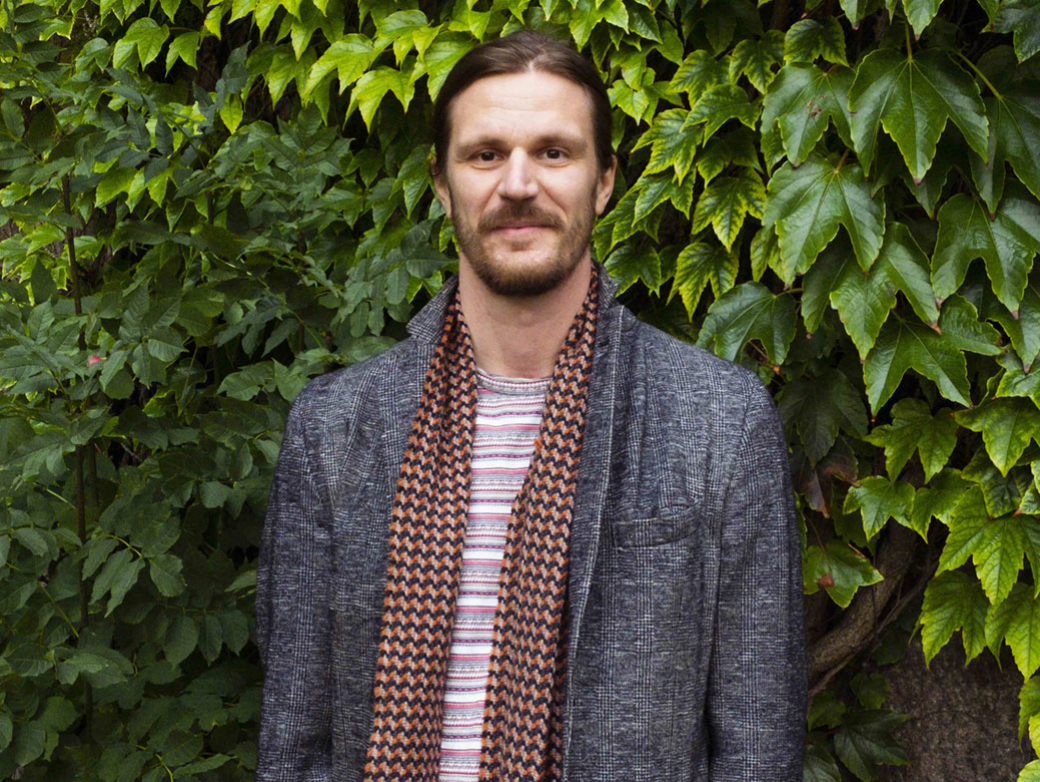


**Andreas Enström**

**Describe your scientific journey and your current research focus**

During my research education, my engagement has been quite broad, where the common denominators are pericytes and hypoxia. More specifically, I am investigating how pericytes communicate with other cells in the brain after hypoxic or ischemic insult and how hypoxia-induced intracellular changes like the induction of RGS5 affect pericyte behaviour, both *in vivo* and *in vitro*.

**Who or what inspired you to become a scientist?**

As a child, I spent a lot of time outdoors and was always curious about exploring the natural world. However, I rather drifted into the field of biology, and it was not until I started university that I became truly passionate about science. I remember several “Aha” moments, for example when reading about Mendelian genetics or stem cell differentiation and knew I wanted to learn more.

**How would you explain the main finding of your paper?**

Cells and tissues are constantly reacting to external factors in order to sense and respond to their environment. For example, a decrease in oxygen levels can put cells under severe stress. Therefore, cells can express certain proteins that orchestrate cellular adaptation to low oxygen levels. One of our main findings is that the protein called RGS5 is rapidly increased under low oxygen conditions in human vascular cells called pericytes. We believe that this increase can be one of the first mechanisms that these cells respond to hypoxia. We also discovered that RGS5 in hypoxic conditions desensitized pericytes to an essential signal mechanism that guides and recruits pericytes to blood vessels where they maintain vessel integrity.

“One of our main findings is that the protein called RGS5 is rapidly increased under low oxygen conditions in human vascular cells called pericytes.”

**What are the potential implications of this finding for your field of research?**

Pathologies linked to acute ischemia/hypoxia are usually associated with a short time-window of treatment to minimize disease progression. Therefore, understanding early mechanisms in how cells respond, and the function of those responses, is important. Our results imply that RGS5 is an early hypoxia-responsive checkpoint in pericytes that could have an important function in regulating downstream events. Although the physiological function of RGS5 after hypoxic onset needs further elucidation, we show that RGS5 regulates chemotactic mechanisms in the presence of the growth factor Platelet-derived growth factor (PDGFBB), which may be a contributing factor to previously shown detrimental effects of RGS5 expression leading to perivascular pericyte loss in e.g. stroke or tumour pathology.

**Figure:**
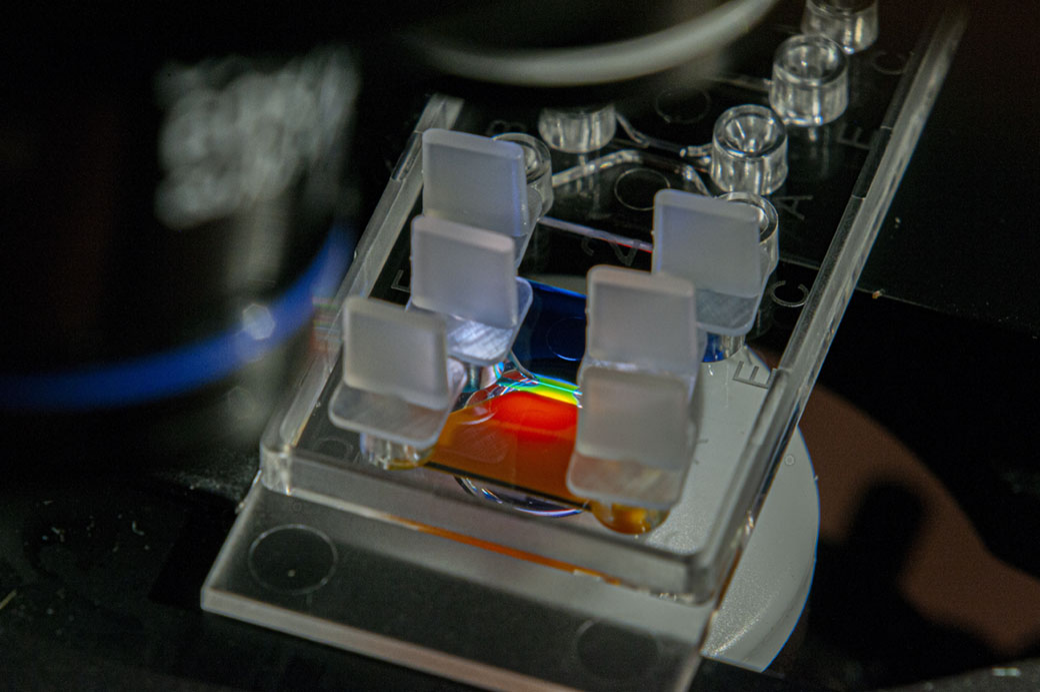
**A microfluidic µ-slide used for studying chemotaxis, where a stable linear concentration gradient is formed in the observation area between the media reservoirs.** For illustrative purposes, this picture was taken using different coloured solutions in each media reservoir.

**Which part of this research project was the most rewarding?**

Due to the short half-life of RGS5 in normoxic conditions, one of the major break throughs was being able to visualize RGS5 on the protein level, which required some optimization of the hypoxic incubation, various protocols and antibody validation.

**What do you enjoy most about being an early-career researcher?**

I greatly appreciate the freedom science gives me. Being in an environment where you can ask a question and then test your hypothesis and discuss your results with friendly and competent colleagues is a great pleasure. Another aspect is the constant problem solving. Science is about getting the closest to the truth as humanly possible. However, many things one may want the answer to are not so easy to observe. Therefore, it is a constant challenge to find ways to gain insight and evaluate your hypothesis. That, at least in part, makes working as a scientist very stimulating.

“I greatly appreciate the freedom science gives me.”

**What piece of advice would you give to the next generation of researchers?**

Try to get a broad understanding early on. There are so many different fields in biology alone and you will most likely feel more passionate about some than others. The same goes for conducting your research. It is worth taking the time to get a good understanding of the available literature before rushing into new projects and wasting valuable time and resources.

**What's next for you?**

If everything goes to plan, I am looking forward to defending my thesis in May of next year.
